# Homeobox Gene *Six3* is Required for the Differentiation of D2-Type Medium Spiny Neurons

**DOI:** 10.1007/s12264-021-00698-5

**Published:** 2021-05-20

**Authors:** Xiaolei Song, Haotian Chen, Zicong Shang, Heng Du, Zhenmeiyu Li, Yan Wen, Guoping Liu, Dashi Qi, Yan You, Zhengang Yang, Zhuangzhi Zhang, Zhejun Xu

**Affiliations:** Institute of Pediatrics, Children’s Hospital of Fudan University, State Key Laboratory of Medical Neurobiology and Ministry of Education Frontiers Center for Brain Science, Institutes of Brain Science, Fudan University, Shanghai, 200032 China

**Keywords:** *Six3*, LGE, Drd2, striatum, Medium spiny neuron

## Abstract

Medium spiny neurons (MSNs) in the striatum, which can be divided into D1 and D2 MSNs, originate from the lateral ganglionic eminence (LGE). Previously, we reported that *Six3* is a downstream target of *Sp8/Sp9* in the transcriptional regulatory cascade of D2 MSN development and that conditionally knocking out *Six3* leads to a severe loss of D2 MSNs. Here, we showed that *Six3* mainly functions in D2 MSN precursor cells and gradually loses its function as D2 MSNs mature. Conditional deletion of *Six3* had little effect on cell proliferation but blocked the differentiation of D2 MSN precursor cells. In addition, conditional overexpression of *Six3* promoted the differentiation of precursor cells in the LGE. We measured an increase of apoptosis in the postnatal striatum of conditional *Six3*-knockout mice. This suggests that, in the absence of *Six3,* abnormally differentiated D2 MSNs are eliminated by programmed cell death. These results further identify *Six3* as an important regulatory element during D2 MSN differentiation.

## Introduction

The basal ganglia consist of several interconnected nuclei, the largest of which is the striatum [1, 2]. Abnormal functions of the striatum are closely associated with Huntington's disease and Parkinson's disease [2–4]. The striatum can be divided into the dorsal and ventral parts [2, 5]. The dorsal striatum comprises the caudate nucleus and putamen, while the ventral striatum includes the nucleus accumbens and olfactory tubercles [6]. Medium spiny neurons (MSNs) constitute as many as 90%–95% of striatal neurons. Also, MSNs can be divided into direct-pathway MSNs, which specifically express dopamine receptor D1 (*Drd1*), and indirect-pathway MSNs, which specifically express dopamine receptor D2 (*Drd2*), according to their axonal projections [7, 8]. Both DRD1- and DRD2-expressing MSNs (D1 and D2 MSNs) have distinct molecular features. For example, D1 MSNs express *Ebf1*, *Isl1*, and *Tac1*, while D2 MSNs specifically express *Adora2a* and *Penk* [8–11].

The ventral lateral ganglionic eminence (vLGE) is the origin of striatal MSNs, while the dorsal LGE (dLGE) mainly generates olfactory bulb interneurons [12, 13]. Based on gene-expression patterns, it has been suggested that the vLGE can be divided into the pLGE3 and pLGE4 (progenitor LGE), and the dLGE can be divided into the pLGE1 and pLGE2 [14]. A variety of transcription factors regulate the development of the LGE. In the dLGE, *Pax6* regulates the development of neurons expressing tyrosine hydroxylase in the olfactory bulb [15, 16]. *Sp8* and *Sp9* are required for the production and survival, as well as the tangential and radial migration of interneurons in the olfactory bulb [12]. In the vLGE, early overexpression of the *Gsx2* gene induces the generation of striatal MSNs. *Gsx2*-null mutants have significantly reduced generation of striatal MSNs [17, 18]. Progenitor cell differentiation is blocked in the LGE subventricular zone (SVZ) of *Dlx1/2* mutant mice [19, 20]. D1 and D2 MSNs are also regulated by specific transcription factors. *Isl1*, *Ebf1*, and *Zfhx3* regulate the development of D1 MSNs [9, 21–24]. Recently, we reported that *Sp8* and *Sp9* are coordinated to regulate D2 MSN generation, differentiation, and survival. *Six3* expression in the LGE is significantly decreased in *Sp8-* and *Sp9-*knockout mice. Accordingly, conditional knockout of *Six3* results in a significant reduction in the number of D2 MSNs, similar to the phenotype in the striatum of *Sp8-* and *Sp9-*knockout mice [25, 26].

The homeobox transcription factor *Six3*, which contains a conserved Six domain and a Six-type homeobox domain, belongs to the *Six* gene family [27]. *Six3* is expressed as early as E6.5–E7.0 at the most anterior region of the embryo and plays important roles in the development of the forebrain and visual system [28–31]. Mutation of *Six3* causes holoprosencephaly [32, 33]. *Six3* is also expressed in ependymal cells, and its dysfunction leads to the inability of ependymal cells to inhibit radial glial activity, which leads to developmental defects of the lateral ventricle wall and abnormal neuroblast migration and differentiation [34]. As a direct downstream target of SP9 and SP8, the transcription factor *Six3* is required for the production of D2 MSNs [26], but the cellular and molecular mechanisms were unknown.

In this study, we investigated the mechanism underlying the effect of a reduction in the number of D2 MSNs in *Dlx5/6-CIE*, *Six3*^flox/flox^ (referred to as *Six3-*cKO) mice. We found that knocked out *Six3* in progenitor cells using *Nestin-Cre* line resulted in severe D2 MSN developmental defect, consistent with the results of *Dlx5/6-CIE* line, but not in *Drd2-Cre* line, which eliminate *Six3* in immature D2 MSNs, indicating *Six3* primarily function in progenitor cells at embryonic stage. The reduction in the number of D2 MSNs in *Six3-*cKO mice mainly ascribe to the abnormal differentiation, but not proliferation defect, of progenitor cells, identifying *Six3* as an important regulatory element of MSN development. These findings broaden our comprehension of the transcriptional mechanisms underlying the development of striatal projection neurons.

## Materials and Methods

### Animals

All experiments were performed in accordance with the National Institutes of Health Guide for the Care and Use of Laboratory Animals and were approved by Animal Ethics Committee of Fudan University. We generated mice that conditionally overexpressed *Six3* by knocking *CAG-promoter*-*Flox-STOP-Flox*-*Six3-IRES-Lacz* into the *Rosa26* locus. *Dlx5/6-CIE* [35], *Nestin-Cre* [36, 37], *Six3* floxed [26, 38], and *Drd2-Cre* mice (from the Mutant Mouse Resource and Research Center) [39] were previously described. Wild-type, *Dlx5/6-CIE*, *Drd2-Cre*; *Six3*^F/+^ and *Six3* floxed littermate mice without the *Cre* allele were used as controls. These mice were on mixed genetic backgrounds of C57BL/6J, 129S6, and CD1. The day on which a vaginal plug was detected was considered embryonic day 0.5 (E0.5), and the day of birth was calculated as postnatal day 0 (P0).

### BrdU Labeling

Pregnant mice were pulsed with 5-bromo-2′-deoxyuridine (BrdU) (50 mg/kg body weight) on E14.5 or E16.5 and embryos were collected and analyzed 30 min after administration.

### Immunohistochemistry

Immunohistochemistry was performed as previously described [26]. Briefly, postnatal and embryonic brains were collected and placed in 4% paraformaldehyde overnight at 4 °C, cryoprotected in 30% sucrose for at least 24 h, frozen in optimal cutting temperature and cryosectioned. All tissues were sectioned coronally at 12 or 20 μm and stained on glass slides.

For SP9, BCL11B, and SIX3 immunohistochemistry, sections were boiled briefly in 10 mmol/L sodium citrate for antigen retrieval. Immunohistochemistry for BrdU^+^ cells was performed after 45 min of incubation in 2 N HCl and rinsing twice in 0.1 mol/L borate buffer at room temperature. Immunofluorescence labeling was performed with the following primary antibodies: rat anti-BCL11B (Abcam, Ab18465), rat anti-BrdU (Accurate Chemical, OBT0030s), chicken anti-β-gal (Abcam, ab9361), rabbit anti-cleaved Caspase-3 (Cell Signaling, #9661), rabbit anti-CRE (Millipore, 69050-3), rabbit anti-EBF1 (Merck, AB10523), rabbit anti-FOXP1 (Abcam, Ab16645), rabbit anti-KI67 (Abcam, ab15580), mouse anti-SIX3 (Santa Cruz Biotechnology, sc-398797), goat anti-SP8 (Santa Cruz Biotechnology, sc-104661), rabbit anti-SP9 [25]. Appropriate Alexa Fluor 488-, Cy3- or Alexa Fluor 647-conjugated secondary antibodies from Jackson ImmunoResearch were used.

### *In situ* RNA Hybridization

*In situ* hybridization was performed on 20-μm cryostat sections as previously described using digoxigenin-labeled riboprobes [25, 26]. Riboprobes were amplified by PCR using the following primers:

*Drd2* forward: CGGGAGCTGGAAGCCTCGA

*Drd2* reverse: TGCAGGGTCAAGAGAAGGCCG

*Adora2a* forward: ATGGGCTCCTCGGTGTACATCATG

*Adora2a* reverse: TCAGGAAGGGGCAAACTCTGAAGAC

*Drd1* forward: ATGGCTCCTAACACTTCTACCATGG

*Drd1* reverse: TCAGGTTGAATGCTGTCCGCTGTG

*Tac1* forward: CCCCTGAACGCACTATCTATTC

*Tac1* reverse: TAGAGTCAAATACCGAAGTCTCAG

*Ebf1* forward: TGACATGAGTCCCAGAGTGGAACTT

*Ebf1* reverse: CACTTCATTCTCCCCTTCCATAGCT

*Isl1* forward: TACGGGATCAAATGCGCCAA

*Isl1* reverse: ACTCAGTACTTTCCAGGGCG

*Six3OS* forward: GGCCGCGCCTTGTAAGCGCTA

*Six3OS* reverse: GTTGAGAATCAGTCTGGGGTCGGC

### Microscopy

Images were captured using an Olympus BX 51 microscope or an Olympus FV1000 confocal microscope system. FV10-ASW software was used to reconstruct the Z-stack confocal images. All images were merged, cropped, and optimized equally using Adobe Photoshop CC.

### Quantification and Statistics

The numbers of *Drd2*-, *Adora2a*-, *Drd1*-*,* and *Tac1*-positive cells in the striatum were counted in 3 randomly-chosen 20-μm sections from each mouse. Three or four control and *Six3* conditional knockout mice from each group were analyzed at P11.

The number of FOXP1^+^ cells and the integrated density of *Ebf1* and *Isl1* measured by ImageJ in the LGE SVZ were quantified in 3 randomly-chosen 12-μm or 20-μm sections from each mouse. Three or four *Dlx5/6-CIE* control and *Six3-*cKO mice from each group were analyzed at E16.5.

The numbers of BCL11B^+^ and BCL11B^+^/EBF1^+^ cells in the striatum was counted in 3 randomly-chosen 12-μm sections from each mouse. Three *Dlx5/6-CIE* control and *Six3-*cKO mice from each group were analyzed at P0.

The number of FOXP1^+^ cells and the integrated density of BCL11B measured by ImageJ in the LGE SVZ were quantified in 3 randomly-chosen 12-μm sections from each mouse, and three *Dlx5/6-CIE* control and *Dlx5/6-CIE*, *Rosa-Six3OE*/+ mice from each group were analyzed at E14.5 and E16.5.

The numbers of BrdU^+^ and KI67^+^ cells in the LGE were counted in 3 randomly-chosen 20-μm sections from each mouse, and 3 control and *Six3-*cKO mice from each group were analyzed at E14.5 or E16.5.

The numbers of SP8^+^ and SP9^+^ cells in the LGE SVZ were counted in 3 randomly-chosen 12-μm sections from each mouse. Three or four *Dlx5/6-CIE* control and *Six3-*cKO mice from each group were analyzed at E14.5 or E16.5. The integrated density of *Six3OS*, *Adora2a*, and *Drd2* measured by ImageJ in the LGE or striatum were quantified in 3 randomly chosen 20 μm sections from each mouse. Three or four *Dlx5/6-CIE* control and *Six3-*cKO mice from each group were analyzed at E16.5 or P0.

The number of cleaved Caspase-3^+^ cells in the striatum was counted in 3 randomly-chosen 20-μm sections from each mouse, and 3 *Dlx5/6-CIE* control and *Six3-*cKO mice from each group were analyzed at P0, P3, P7, and P11.

Statistical significance was determined using unpaired Student’s *t*-test (*P < 0.05, **P < 0.01, and ***P < 0.001). The results are presented as the mean + SEM.

## Results

### *Six3* Mainly Functions in D2 MSN Precursor Cells

**Fig. 1:**
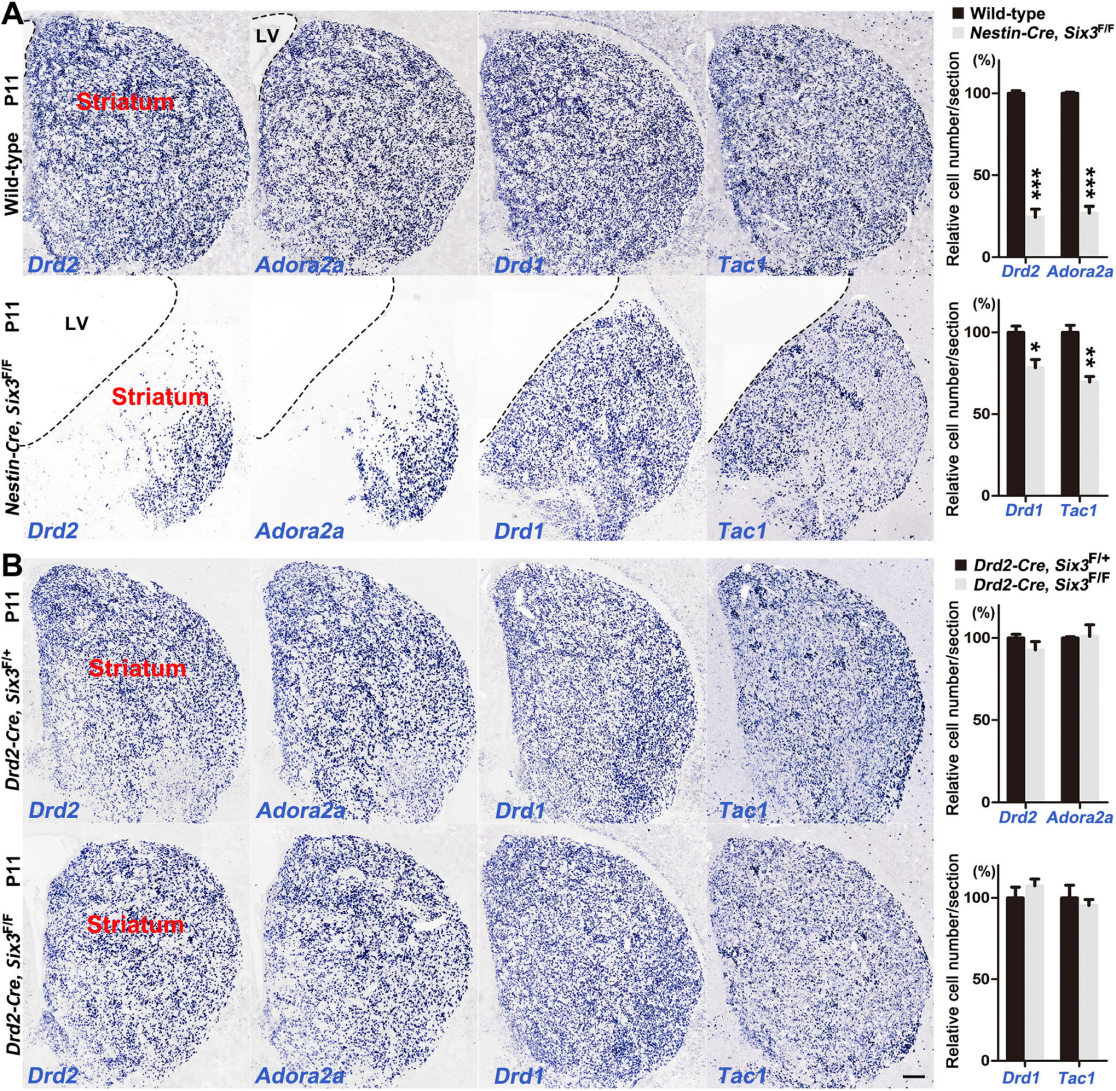
*Six3* mainly functions in D2 MSN precursor cells. **A** Left panels, *in situ* RNA hybridization for *Drd2*, *Adora2a*, *Drd1* and *Tac1* in the striatum of wild-type control and *Nestin-Cre*, *Six3*^F/F^ mice at P11. Note that most of the *Drd2*^*+*^ and *Adora2a*^*+*^ cells in the medial LGE of *Nestin-Cre*, *Six3*^F/F^ mice were lost. Right panel, quantification of *Drd2*, *Adora2a*, *Drd1* and *Tac1* (*n* = 3–4). The dotted lines indicate the border of the lateral ventricle (LV) and striatum. **B** Left panels, *in situ* hybridization for *Drd2*, *Adora2a*, *Drd1* and *Tac1* in the striatum of control and *Drd2-Cre*, *Six3*^F/F^ mice at P11. Note that the development of both D1 and D2 MSNs was unaffected in the striatum of *Drd2-Cre, Six3*^F/F^ mice compared to the controls (*Drd2-Cre, Six3*^F/+^). Right panel, quantification of *Drd2*, *Adora2a*, *Drd1*, and *Tac1* (*n* = 3–4). Data shown are the mean + SEM (*P < 0.05, **P < 0.01, ***P < 0.001, Student’s *t*-test; scale bar, 200 μm).

We previously reported that the number of striatal D2 MSNs is significantly decreased in *Six3* conditional knockout mice [26]. We crossed *Six3* floxed mice with *Nestin-Cre* and *Drd2-Cre* lines to further examine the function of *Six3* (Fig. 1). According to our previously published paper, SIX3 is prominently expressed in the pLGE3 domain of the SVZ and scattered in the LGE mantle zone (MZ) at the embryonic stage. Its expression is later than that of *Sp9* but earlier than that of *Drd2-EGFP*. The expression of *Six3* is rapidly down-regulated in the striatum at the postnatal stage [26]. The *Nestin-Cre* line, in which *Six3* was knocked out earlier than in *Dlx5/6-CIE* mice, expressed CRE protein in neural stem cells in the LGE [40–42], whereas in the *Drd2-Cre* line, *Six3* was deleted in immature D2 MSNs after it was expressed for a short time, as *Drd2* was expressed later than *Six3* in the LGE [26]. At P11, the volume of the lateral ventricles was increased while that of the striatum was reduced in *Nestin-Cre*, *Six3*^F/F^ mice compared with wild-type control mice (Fig. 1A). This is consistent with our previous results [26]. The numbers of *Drd2*^+^ and *Adora2a*^+^ MSNs in the striatum of *Nestin-Cre*, *Six3*^F/F^ mice were greatly decreased compared with those in control mice. The reductions of *Drd2*^+^ and *Adora2a*^+^ MSNs mainly occurred in the medial striatum, indicating that the generation of late-born D2 MSNs was compromised in *Nestin-Cre*, *Six3*^F/F^ mice (Fig. 1A). The numbers of *Drd1*^+^ and *Tac1*^+^ cells, though significantly decreased, were relatively less affected (Fig. 1A). These decreased *Drd1*^+^ and *Tac1*^+^ cells might be ascribed to an ependymal cell defect since the development of ependymal cells was disturbed in *Nestin-Cre*, *Six3*^F/F^ mice [34]. In contrast, *Drd2*^+^, *Adora2a*^+^ and *Drd1*^+^, and *Tac1*^+^ MSNs were densely distributed in the striatum of both *Drd2-Cre*, *Six3*^F/F^ mice and *Drd2-Cre*, *Six3*^F/+^ control mice at P11 (Fig. 1B). The numbers of both D1 and D2 MSNs were comparable in *Drd2-Cre*, *Six3*^F/F^ mice and *Drd2-Cre*, *Six3*^F/+^ control mice at P11 (Fig. 1B). This indicates that the development of D2 MSNs is unaffected when *Six3* function is blocked in immature D2 MSNs. These results suggest that *Six3* mainly functions before D2 MSNs differentiate and gradually loses its function as they mature.

### D2 MSN Neurogenesis is Reduced in the LGE SVZ of* Six3*-cKO Mice

**Fig. 2:**
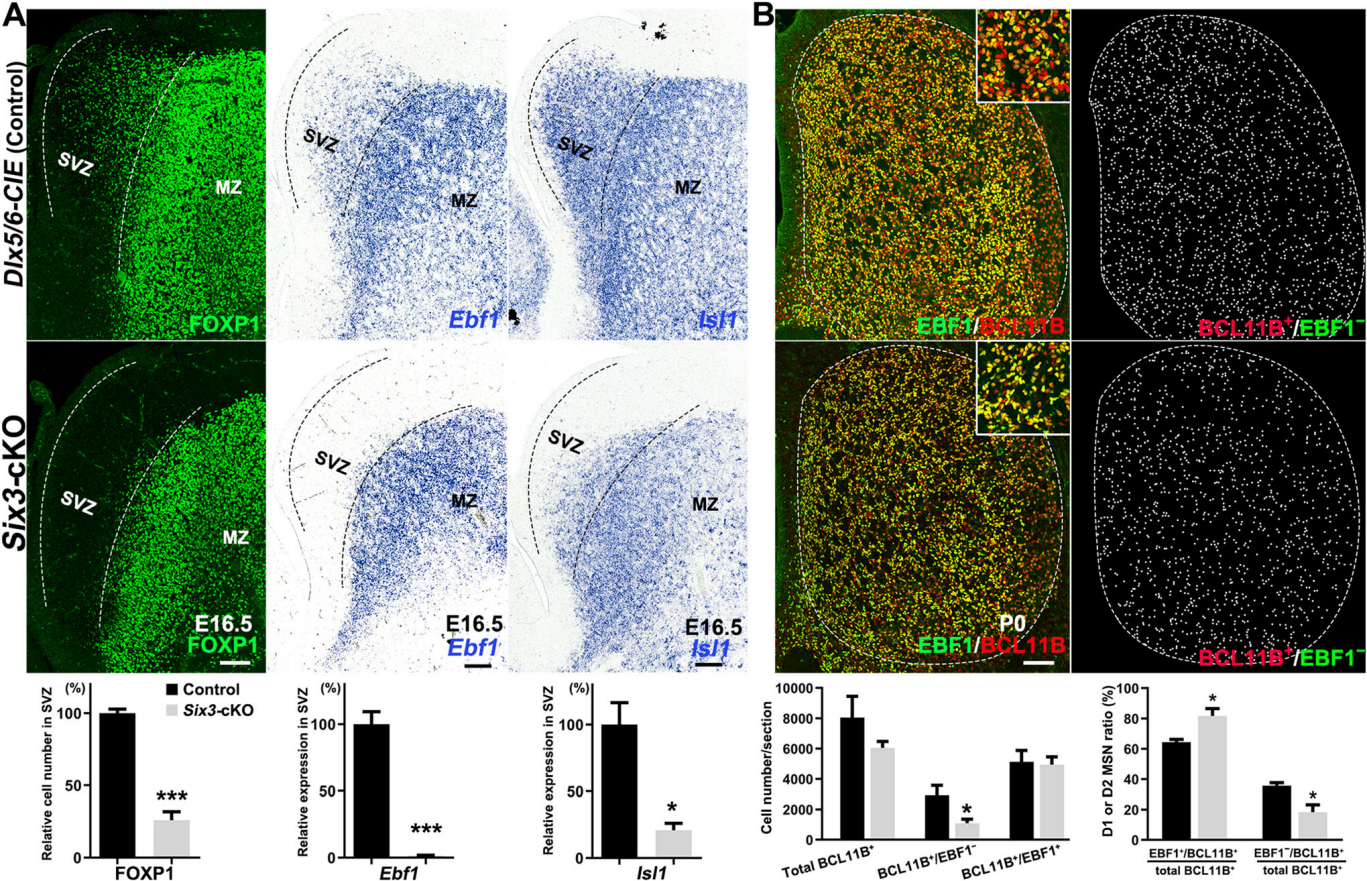
Neurogenesis is reduced in the LGE of *Six3*-cKO mice. **A** Upper panels. FOXP1 immunofluorescence and *Ebf1* and *Isl1 in situ* hybridization in the LGE of control and *Six3-*cKO mice at E16.5. The LGE SVZ of *Six3-*cKO mice contains fewer FOXP1^+^, *Ebf1*^+^ and *Isl1*^+^ cells than those of controls. The dotted lines indicate the border of the LGE SVZ and MZ. Lower panels, quantification of FOXP1, *Ebf1*, and *Isl1*. Data shown are the mean + SEM (*n* = 3–4; *P < 0.05, ***P < 0.001, Student’s *t*-test). **B** Upper panels, BCL11B and EBF1 immunofluorescence in the striatum of control and *Six3-*cKO mice at P0. BCL11B^+^/EBF1^+^ cells represent D1 MSNs, and BCL11B^+^/EBF1^−^ cells represent D2 MSNs. Inserts show magnified images of BCL11B and EBF1 co-expression in control and *Six3-*cKO mice. BCL11B^+^/EBF1^−^ cells (D2 MSNs) are indicated by white dots in the right panel. Lower left panel, quantification showing that the number of BCL11B^+^/EBF1^−^ cells, but not BCL11B^+^/EBF1^+^ cells, was significantly lower in the striatum of *Six3-*cKO mice than in control. Lower right panel, percentages of (BCL11B^+^/EBF1^+^)/BCL11B^+^ and (BCL11B^+^/EBF1^−^)/BCL11B^+^ cells. Dotted line indicates the striatal border. Data shown are the mean + SEM (*n* = 3; *P < 0.05, Student’s *t*-test; scale bar, 200 μm).

To investigate the cause of the significant reduction in the number of striatal D2 MSNs in the absence of *Six3*, we further examined neurogenesis in the striatum of *Six3*-cKO and *Dlx5/6-CIE* control mice. The LGE SVZ at later developmental stages contains proliferating cells and differentiated cells [43]. FOXP1^+^ cells in the LGE SVZ are differentiated newborn MSNs [44]. We found that the expression of FOXP1 in the LGE SVZ was severely reduced in *Six3*-cKO mice compared to control mice at E16.5 (Fig. 2A), indicating a reduction of neurogenesis in both D1 and D2 MSNs. Consistent with this result, we found that the D1 MSN-specific marker *Ebf*1 [9] was significantly reduced in the LGE SVZ of *Six3-*cKO mice at E16.5 (Fig. 2A). The expression of *Isl1*, another D1 MSN-specific marker [9], was also greatly reduced in the LGE SVZ of *Six3-*cKO mice compared to that in control mice at E16.5 (Fig. 2A).

We next examined the number of striatal MSNs at P0 using BCL11B, a pan-striatal MSN marker, combined with EBF1 expression to distinguish D1 MSNs from D2 MSNs [9, 45]. BCL11B^+^/EBF1^+^ cells represented D1 MSNs, BCL11B^+^ and EBF1 immuno-negative (BCL11B^+^/EBF1^−^) cells represented D2 MSNs (Fig. 2B). We found no significant difference in the number of EBF1^+^ cells (D1 MSNs), but the number of BCL11B^+^/EBF1^−^ cells (D2 MSNs) was significantly lower in *Six3-*cKO mice than in controls at P0 (Fig. 2B). These results suggest that neurogenesis of D1 MSNs is reduced at the embryonic stage, but not at the postnatal stage. This indicates that the absence of *Six3* results in a subpopulation of D1 MSNs with delayed differentiation at the embryonic stage. In contrast, the neurogenesis of D2 MSNs was significantly decreased in the LGE of *Six3-*cKO mice from the embryonic to the postnatal stage (Figs 1A and 2).

### Cell Proliferation is Unaffected in the LGE of* Six3*-cKO Mice

**Fig. 3:**
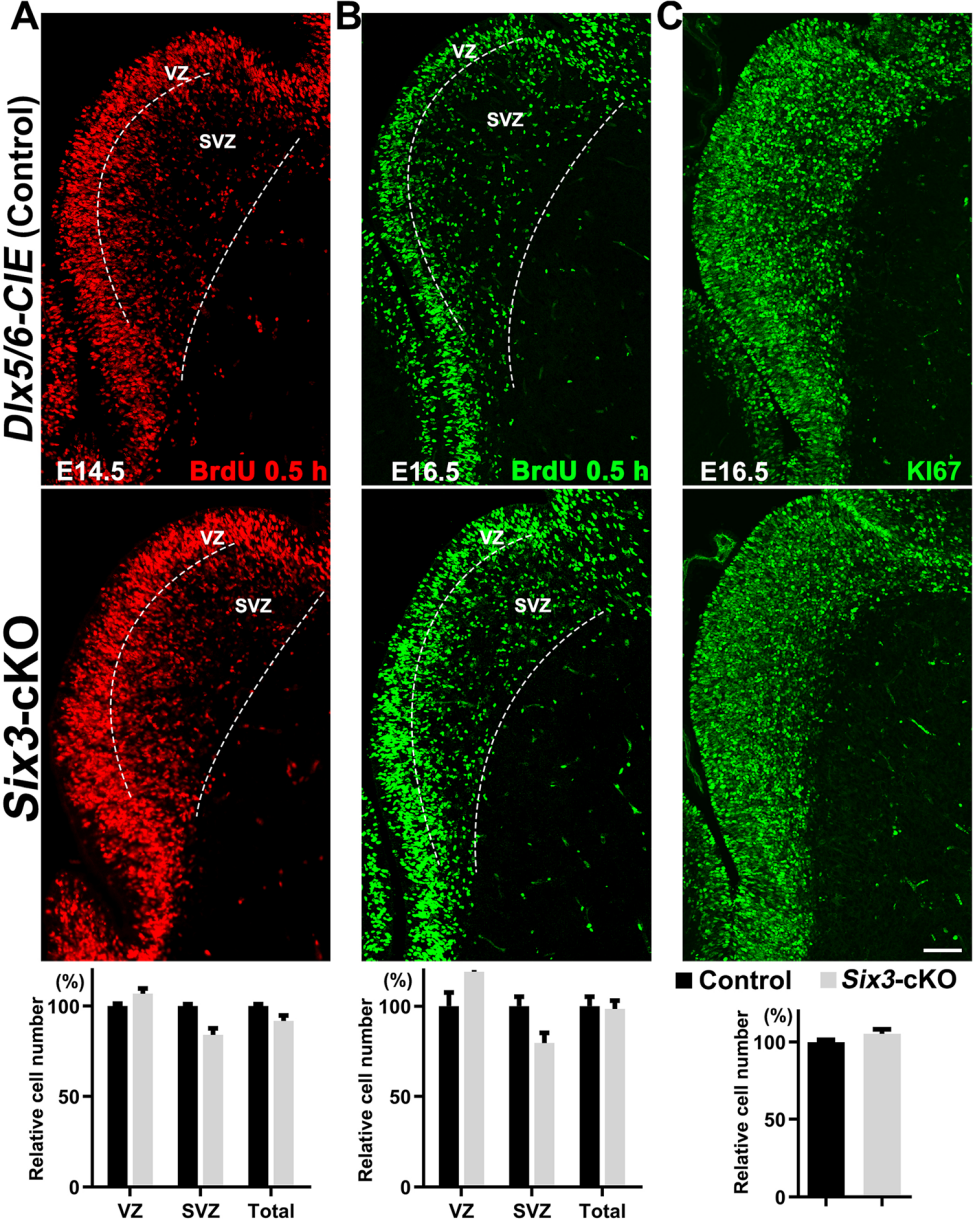
Cell proliferation is unaffected in *Six3*-cKO mice. **A, B** Upper panels, immunofluorescence images showing BrdU-pulse labeling for 0.5 h in the LGE of control and *Six3-*cKO mice at E14.5 (**A**) and E16.5 (**B**). Lower panels, numbers of BrdU^+^ cells in the LGE VZ and SVZ at E14.5 (**A**) and E16.5 (**B**). There is no significant difference in the number of BrdU^+^ cells between the LGEs of *Six3-*cKO and control mice. **C** Upper panels, KI67 immunofluorescence in the LGE of control and *Six3-*cKO mice at E16.5. Dotted lines indicate the border of the LGE VZ, SVZ, and MZ. Lower panel, quantification data (mean + SEM, *n* = 3, Student’s *t*-test; scale bar, 100 μm).

Next, we determined whether LGE cell proliferation was changed in *Six3-*cKO mice, as a small population of SIX3^+^ cells were in S-phase, and *Six3* has been reported to regulate cell proliferation [46, 47]. It is possible that *Six3* functions by regulating the cell cycle during LGE development. We performed 30-min BrdU pulse-labeling experiments at E14.5 and E16.5 (Fig. 3A, B). The number of BrdU^+^ cells was comparable in *Six3-*cKO and control mice at E14.5 (Fig. 3A). Consistent with this, the data showed that the total number of BrdU^+^ cells in the LGE was also unchanged in the LGE of *Six3-*cKO mice compared to that of control mice at E16.5 (Fig. 3B). Notably, BrdU^+^ cells seemed to accumulate in the ventricular zone (VZ) at E14.5 and E16.5, since we saw more BrdU^+^ cells (but no significant difference) in the VZ of *Six3-*cKO mice (Fig. 3B). Because *Dlx5/6-CIE* mice expressed little CRE in the VZ, we proposed that the slight accumulation of BrdU^+^ cells in the VZ was a secondary effect of the blocked differentiation of progenitor cells in the LGE SVZ. We also found that the expression of KI67, a classical cell proliferation marker, in the LGE was slightly but not significantly higher in *Six3-*cKO mice than that in control mice (Fig. 3C). These results suggest that cell proliferation during LGE development is little affected in the absence of *Six3* function.

### Differentiation of D2 MSN Precursor Cells is Blocked in* Six3*-cKO Mice

**Fig. 4:**
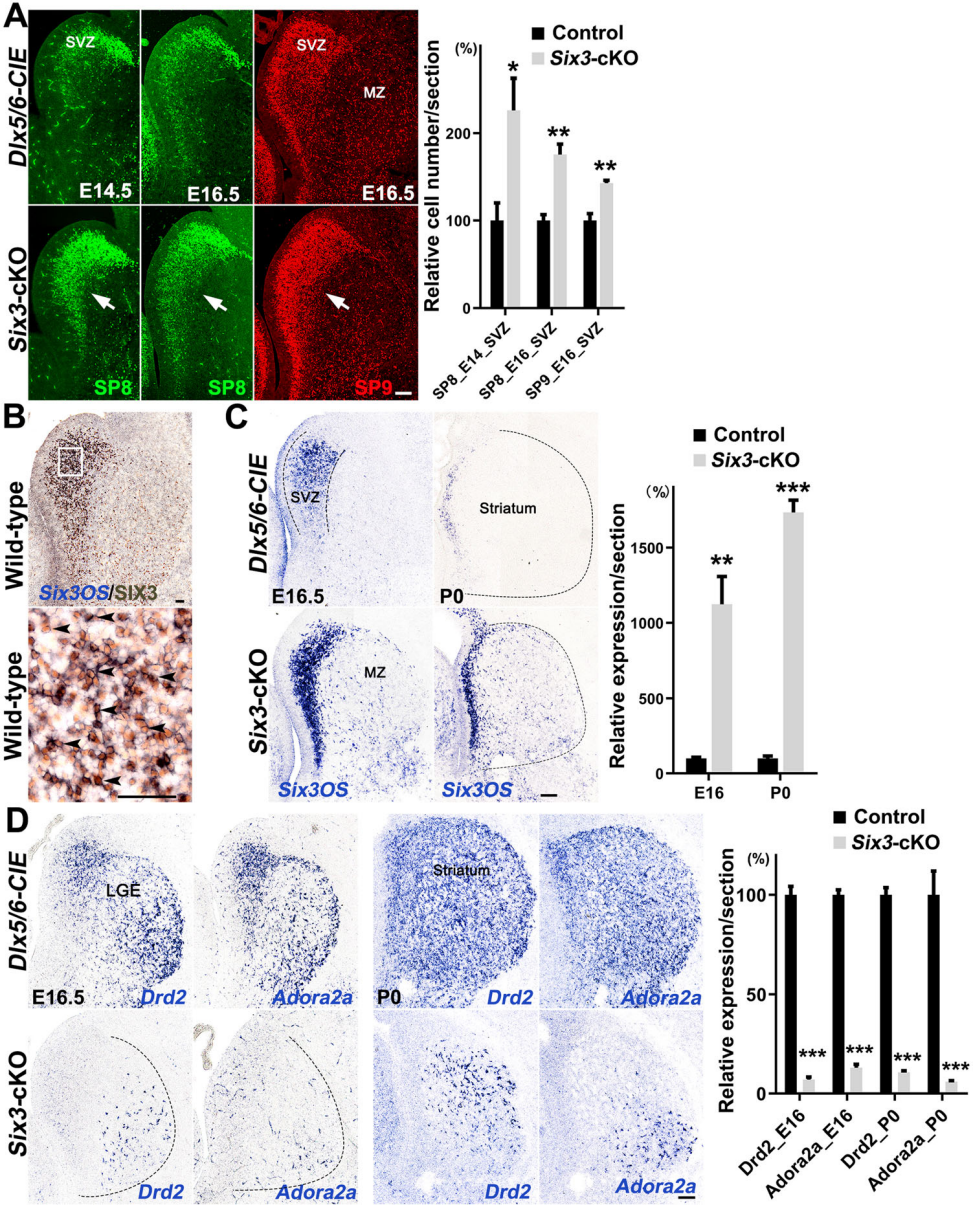
Differentiation of striatal D2 MSNs is blocked in *Six3*-cKO mice. **A** Left panels, immunofluorescence images showing SP8 (E14.5 and E16.5) and SP9 (E16.5) expression in the LGE. Arrows indicate that SP8^+^ and SP9^+^ cells are accumulated in the vLGE SVZ of *Six3-*cKO mice compared to controls. Right panel, quantification data is shown (*n* = 3–4). **B** Left panels, immunohistochemistry images showing SIX3 protein and *Six3OS* mRNA expression in the LGE of wild-type mice at E16.5. The magnified image shows that most, if not all, of the *Six3OS*^+^ cells in the LGE SVZ express the SIX3 protein. Right panels, quantification. **C** Left panels, *in situ* hybridization for *Six3OS* in the LGE of control and *Six3-*cKO mice at E16.5 and P0. *Six3OS* is mainly expressed in the LGE SVZ, but the expression of *Six3OS* is greatly increased in the LGE of *Six3-*cKO mice. Note that many *Six3OS*^+^ cells are distributed in the LGE MZ and striatum of *Six3-*cKO mice. Right panel, quantification data (*n* = 3–4). **D** Left panels, *in situ* hybridization for *Drd2* and *Adora2a* in control and *Six3-*cKO mice at E16.5 and P0. *Drd2 and Adora2a* are strongly expressed in the controls at both E16.5 and P0. However, very little *Drd2* and *Adora2a* mRNA is expressed in the LGE and striatum of *Six3-*cKO mice at E16.5 and P0. Right panel, quantification data (*n* = 3; mean + SEM; *P < 0.05, **P < 0.01, ***P < 0.001, Student’s *t*-test). Note that many SP9^+^ cells, BCL11B^+^/EBF1^−^ cells, and *Six3OS*^+^ cells are located in the LGE MZ and striatum of *Six3-*cKO mice in **A** and **B**. Dotted lines indicate the borders of the LGE. Scale bars, 100 μm in **A**, **C**, and **D**; 50 μm in **B**.

Since cell proliferation was not compromised in *Six3-*cKO mice, we hypothesized that the significant loss of D2 MSNs was due to abnormal differentiation of precursor cells. We assessed *Sp8* and *Sp9* expression in the LGE as they are expressed earlier than *Six3* and are upstream of it in the LGE SVZ [26]. Normally, cells with high SP8 expression were located in the dLGE SVZ, while cells with low expression level of SP8 were located in the vLGE SVZ at E14.5 and E16.5 (Fig. 4A). SP9^+^ cells located in the SVZ were mainly precursor cells, and those in the MZ were mainly D2 MSNs [25]. SP8^+^ cells were significantly higher in the vLGE SVZ in *Six3-*cKO embryos than in controls at E14.5 and E16.5 (Fig. 4A). The number of SP9^+^ cells was also significantly increased in the LGE SVZ of *Six3-*cKO embryos at E16.5 (Fig. 4A). The increased number of SP8^+^ and SP9^+^ cells in the LGE SVZ indicated that MSN precursor cells accumulated in *Six3-*cKO embryos. Although SP8^+^ and SP9^+^ precursor cells accumulated in the LGE SVZ, we still observed many SP9^+^ cells in the LGE MZ of *Six3-*cKO embryos (Fig. 4A). Because SP9 is mainly expressed in D2 MSNs in the striatum [25], these SP9^+^ cells in the LGE MZ of *Six3-*cKO embryos were putative D2 MSNs.

As described above, BCL11B^+^/EBF1^−^ cells in the striatum were visualized as white dots (Fig. 2B). The numbers of BCL11B^+^/EBF1^−^ cells was lower in *Six3-*cKO mice than in controls (Fig 2B), but there were still many BCL11B^+^/EBF1^−^ cells in the striatum (Fig. 2B). This also indicated that putative D2 MSNs were generated in the LGE MZ of *Six3-*cKO mice, consistent with above result (Fig 4A). Besides, the ratio of D1 MSNs was increased while that of D2 MSNs was decreased in *Six3*-cKO mice, indicating that losing *Six3* function in LGE leads to abnormal neural differentiation.

*Six3OS* has been reported to be co-expressed with *Six3* in the hypothalamus and retina to indirectly regulate the function of *Six3* [48, 49]. We immunostained for SIX3 after *in situ* hybridization for *Six3OS* mRNA, and found that most, if not all, *Six3OS*^+^ cells expressed SIX3 (Fig. 4B). This indicated that *Six3OS* and SIX3 are expressed in the same cell type in the LGE SVZ. *Six3OS* was strongly expressed in the LGE SVZ but seldom in the MZ of control mice (Fig. 4C), suggesting that *Six3OS* is mainly expressed in precursor cells. *Six3OS* expression was significantly up-regulated in the LGE SVZ of *Six3-*cKO mice (Fig. 4C), indicating that *Six3OS*^+^ precursor cells accumulate in the LGE SVZ in the absence of *Six3*. Surprisingly, we also found that many *Six3OS*^+^ cells were located in the LGE MZ or striatum at E16.5 and P0, suggesting that *Six3OS* expression in those precursor cells is not down-regulated and they then migrated into the MZ or striatum with insufficient maturation after loss of *Six3* (Fig. 4C). Once again, *Six3OS*^+^ cells in the MZ indicated that *Six3OS*^+^ putative D2 MSNs are generated in the LGE of *Six3-*cKO mice, consistent with the above results.

We then examined the expression of the differentiated D2 MSN markers *Drd2* and *Adora2a* at E16.5 and P0 (Fig. 4D). *Drd2* and *Adora2a* were strongly expressed in the LGE SVZ and MZ in control mice (Fig. 4D). However, only a few cells expressing high levels of *Drd2* and very few *Adora2a*^+^ cells were located in the LGE MZ of *Six3-*cKO mice at E16.5 (Fig. 4D). Similarly, *Drd2* and *Adora2a* were strongly expressed in the striatum of control mice but significantly reduced in that of *Six3-*cKO mice at P0 (Fig. 4D). We previously reported that the cells that express *Drd2* strongly are mainly striatal cholinergic interneurons [25, 26]. Thus, SP8^+^ and SP9^+^ precursor cells accumulated in the LGE SVZ, SP9^+^ cells and many BCL11B^+^/EBF1^−^ putative D2 MSNs located in the LGE MZ and striatum, but few *Drd2*^+^ and *Adora2a*^+^ cells were found in the LGE and striatum, providing strong evidence that D2 MSN precursor cells differentiate abnormally in the striatum without *Six3*.

### Overexpression of* Six3* Promotes the Differentiation of MSN Precursor Cells

**Fig. 5:**
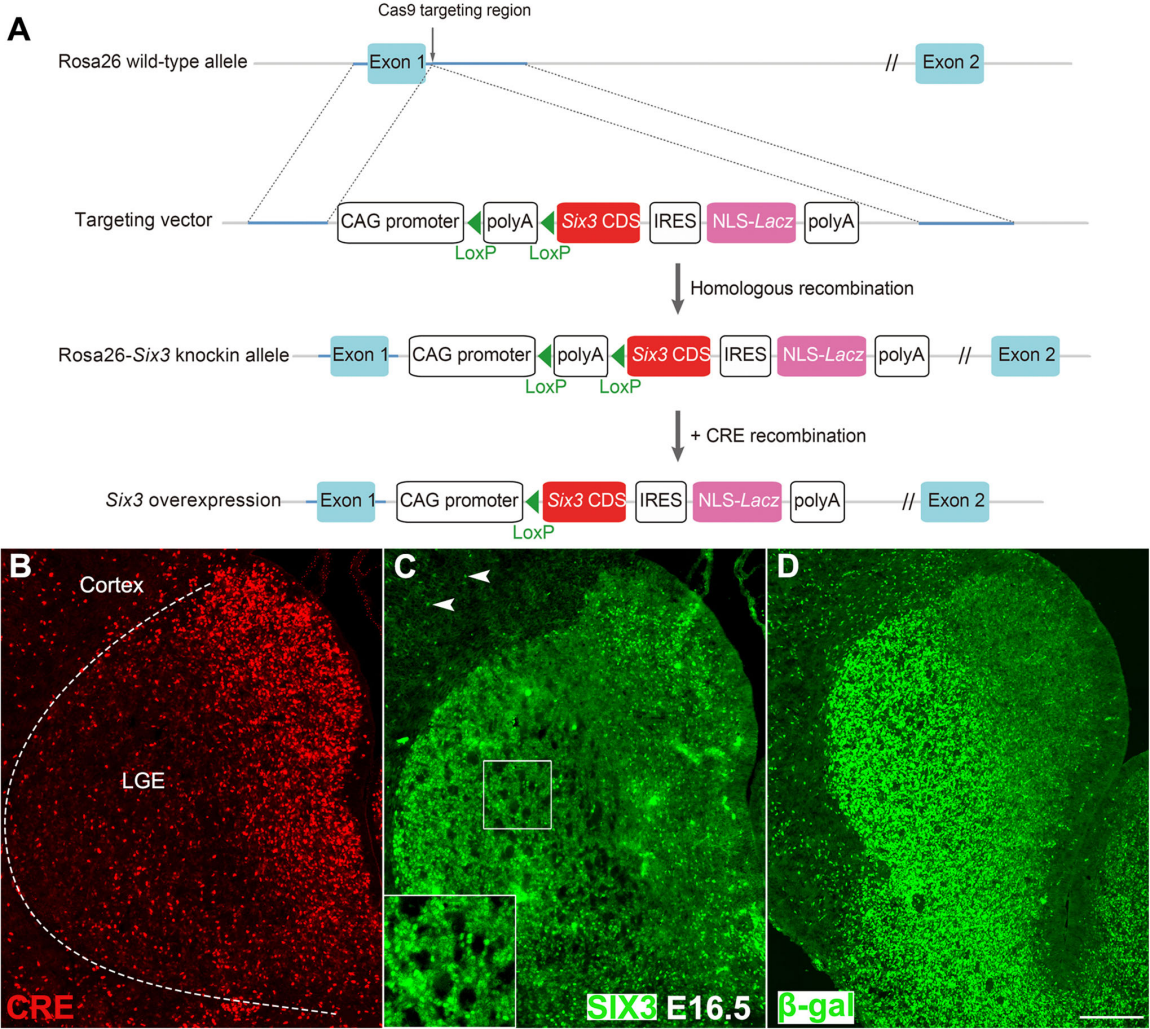
Generation of mice with conditional overexpression of *Six3*. **A** A cassette containing the *CAG promoter*-*Flox-STOP-Flox*-*Six3-IRES-Lacz* sequence was knocked into the downstream of exon 1 of *Rosa26* (gene trap ROSA 26). The *Lacz* gene contains a nuclear localization sequence (NLS). **B–D** Immunofluorescence images showing CRE, SIX3, and β-galactosidase (β-gal) expression in *Dlx5/6-CIE*, *Rosa26-Six3OE/+* mice at E16.5. Arrowheads in **C** show ectopic expression of SIX3 in Dlx5/6^+^ cells in the cortex. Boxes show magnified images of SIX3 expression in the LGE MZ (scale bar, 200 μm).

To investigate the differentiation action of *Six3*, we generated a mouse line that conditionally overexpressing *Six3* by knocking *CAG promoter*-*Flox-STOP-Flox*-*Six3-IRES-Lacz* into the *Rosa26* locus (*Rosa-Six3OE* allele) and using *Dlx5/6-CIE* to drive continuous *Six3* expression (Fig. 5A). SIX3 was expressed in almost all of the MSNs in *Dlx5/6-CIE*, *Rosa-Six3OE*/+ mice, and the expression of β-galactosidase, with a nuclear localization sequence, confirmed this phenotype (Fig. 5B–D). Accordingly, SIX3^+^ cells were distributed in the cortices of *Dlx5/6-CIE*, *Rosa-Six3OE*/+ mice (Fig. 5B), although *Six3* is not normally expressed in the cortex [26]. These results demonstrated that *Six3* was ectopically overexpressed in the *Dlx5/6-CIE*, *Rosa-Six3OE*/+ mice.

**Fig. 6:**
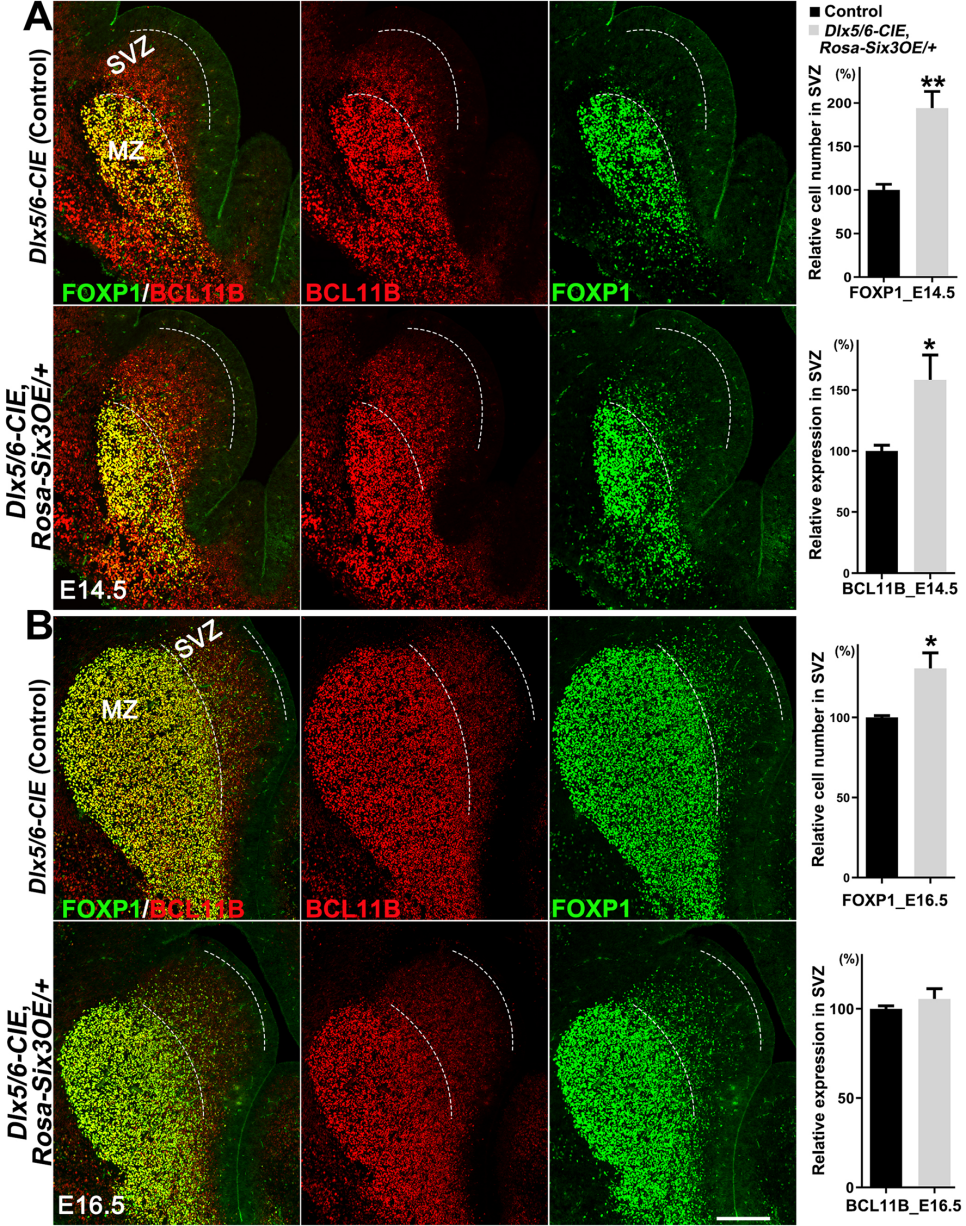
Overexpression of *Six3* in the LGE promotes the differentiation of MSN precursor cells.** A** Left, immunofluorescence images showing FOXP1 and BCL11B expression in the LGE of control and *Dlx5/6-CIE, Rosa-Six3OE/+* mice at E14.5. Note that there are very few FOXP1^+^ cells in the LGE SVZ of controls. BCL11B expression is up-regulated in the LGE SVZ of *Dlx5/6-CIE, Rosa-Six3OE/+* mice compared to controls. Right, quantification showing that the number of FOXP1^+^ cells in the LGE SVZ is significantly higher in *Dlx5/6-CIE, Rosa-Six3OE/+* mice than in controls. **B** Left, immunofluorescence images showing FOXP1 and BCL11B expression in the LGE of control and *Dlx5/6-CIE, Rosa-Six3OE/+* mice at E16.5. BCL11B expression is higher in the LGE SVZ of *Dlx5/6-CIE, Rosa-Six3OE/+* mice than in controls. Right, quantification data (*n* = 3; mean + SEM; *P < 0.05, **P < 0.01, Student’s *t*-test; scale bar, 200 μm).

We used BCL11B and FOXP1 to assess whether *Six3* overexpression promotes precursor cell differentiation (Fig. 6). Both BCL11B and FOXP1 were weakly expressed in the LGE SVZ but strongly expressed in the MZ of *Dlx5/6-CIE* control mice (Fig. 6). We found that the expression of BCL11B was significantly increased in the LGE SVZ of *Six3* conditional overexpression mice at E14.5 (Fig. 6A). Similarly, very few FOXP1^+^ cells were located in the LGE SVZ of control mice, but many FOXP1^+^ cells were distributed in the LGE SVZ of *Six3* conditional overexpression mice at E14.5 (Fig. 6A). The quantification confirmed that the number of FOXP1^+^ cells was significantly higher in the LGE SVZ of *Six3* conditional overexpression mice than in controls (Fig. 6A). We also analyzed BCL11B and FOXP1 expression at E16.5 (Fig. 6B). The expression of BCL11B in the LGE SVZ of *Dlx5/6-CIE*, *Rosa-Six3OE*/+ mice was slightly up-regulated compared to controls (Fig. 6B). We confirmed this phenotype by counting the FOXP1^+^ cells in the LGE SVZ and found that the number of these cells was significantly increased in *Dlx5/6-CIE*, *Rosa-Six3OE*/+ mice (Fig. 6B). These results suggested that *Six3* promotes precursor cell differentiation in the LGE.

### Loss of* Six3* Induces Apoptosis in the Postnatal Striatum

**Fig. 7:**
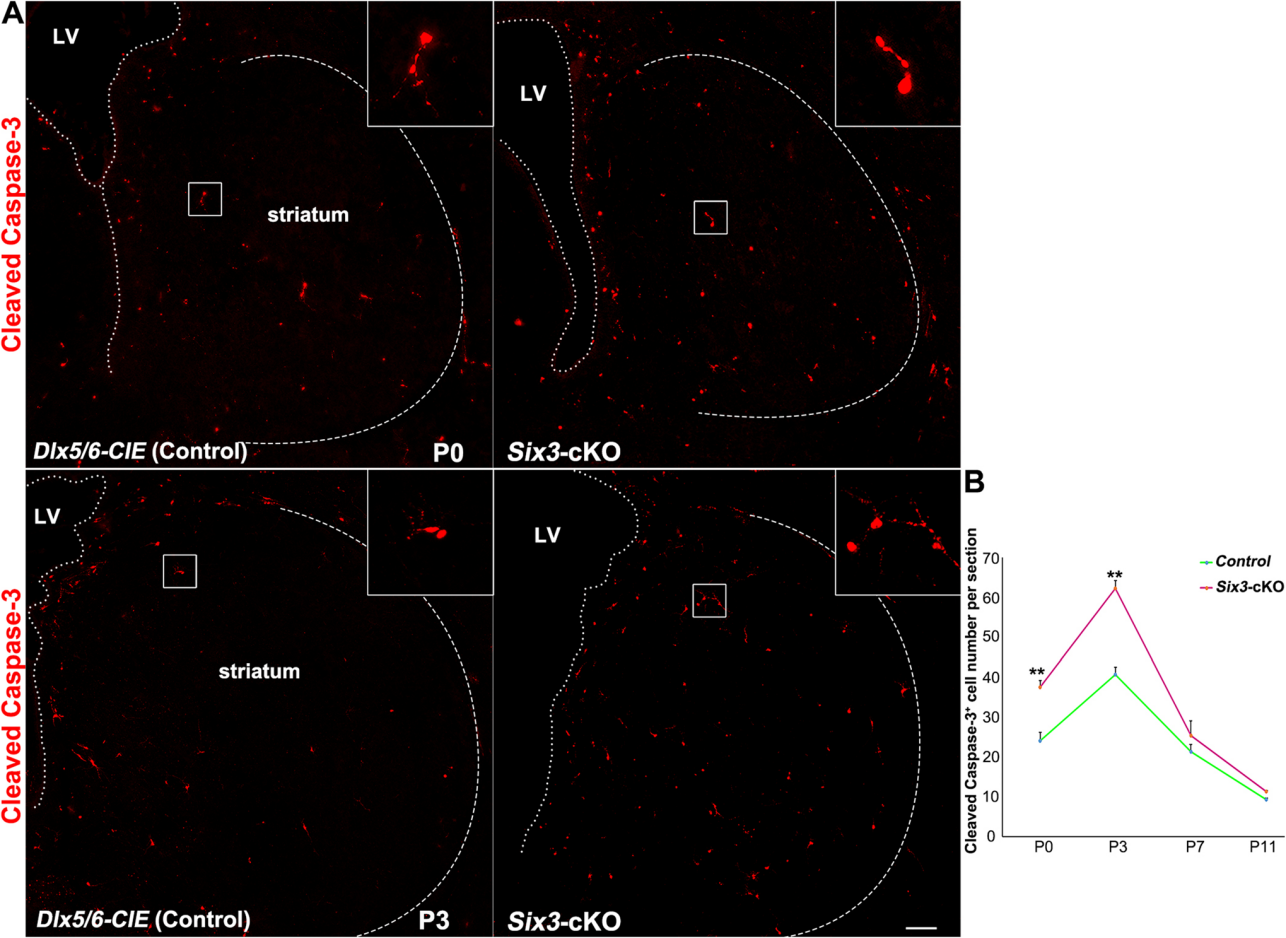
Apoptosis is increased in the striatum of postnatal *Six3-*cKO mice. **A** Immunofluorescence images showing cleaved Caspase-3 expression in the striatum of *Dlx5/6-CIE* controls and *Six3-*cKO mice at P0 and P3 (dotted lines, border of the striatum; inserts, magnified images of cleaved Caspase-3). **B** Quantification showing the number of cleaved Caspase-3^+^ cells was significantly higher in *Six3-*cKO mice than in control at P0 and P3, while there was no significant difference at P7 and P11 (*n* = 3; mean + SEM; **P < 0.01, Student’s *t*-test; scale bar, 100 μm).

We analyzed cell death by evaluating cleaved Caspase-3 expression to investigate whether abnormally differentiated D2 MSNs survived in the striatum of postnatal *Six3-*cKO mice. The data showed that in control mice, the number of Caspase-3^+^ cells increased from P0, peaked at P3, and then decreased to a very low level as the striatum developed (Fig. 7). Without *Six3* function, we found that the number of Caspase-3^+^ cells was significantly higher than in controls at P0 and P3, but that there was no significant difference at P7 or P11 (Fig. 7). We inferred that most of the dying cells were abnormally-differentiated D2 MSN precursor cells and that these cells were finally eliminated by programmed cell death, as we did not find a severe reduction in the number of D1 MSNs in *Six3-*cKO mice (Fig. 1).

## Discussion

Striatal MSNs originate from the vLGE, while olfactory bulb interneurons are generated in the dLGE [13]. The LGE VZ was subdivided into 4 domains according to the combinatorial expression of several transcription factors. *Pax6*, *Gsx2*, and *Er81* (*Etv1*) were used to identify the dLGE. The vLGE contained the pLGE3, in which *Isl1* was strongly expressed while there was no expression of *Er81,* and the pLGE4, in which *Nkx6.2* was strongly expressed while there was little expression of *Isl1*. The development of the two types of MSNs is regulated by many transcription factors [14]. For example, *Gsx2*, *Ascl1*, and *Dlx1/2* are required for pan-striatal MSN development [50]; *EBf1* and *Isl1* specifically regulate D1 MSN differentiation and axonal guidance [9, 21–23]; *Sp8* and *Sp9* regulate D2 MSN generation and survival [25, 26]. Here, we found that *Six3* is required for D2 MSN differentiation and that its function is gradually reduced during D2 MSN maturation.

*Six3* is expressed in ependymal cells of postnatal mice, and is essential for the maturation of ependymal cells. In mice in which *Six3* is conditionally knocked out by *Nestin-Cre*, cells located in the lateral ventricle wall contain mixed characteristics of ependymal cells and radial glia [34]. These defective cells result in the abnormal migration and differentiation of neuroblasts, markedly enlarged lateral ventricles, and hydrocephalus [34]. In this study, we obtained the same results, i.e., a reduction in the number of D2 MSNs and enlarged lateral ventricles, in both *Dlx5/6-CIE, Six3*^F/F^ and *Nestin-Cre, Six3*^F/F^ mice. The *Six3* gene was deleted in ependymal cells in *Nestin-Cre*, *Six3*^F/F^ mice, but not in *Dlx5/6-CIE*, *Six3*^F/F^ mice, as *Dlx5/6* were rarely expressed in the progenitors (neural stem cells) of ependymal cells. However, the lateral ventricles of both *Dlx5/6-CIE, Six3*^F/F^ and *Nestin-Cre, Six3*^F/F^ mice were significantly enlarged, consistent with our previous report on *Sp9* mutant mice [25, 26]. This might indicate that enlargement of the lateral ventricle in *Nestin-Cre*, *Six3*^F/F^ mice is caused by the significant reduction in the number of D2 MSNs in the striatum.

It has been reported that *Isl1*, the conditional knockout of which results in a significant reduction in the number of D1 MSNs, regulates the development of D1 MSNs through semaphorin 3E (*Sema3e*) signaling, and that *Ebf1* also regulates the differentiation of D1 MSNs [9, 21, 22]. We previously showed that few ISL1^+^ or EBF1^+^ cells express the SIX3 protein [26]. In this study, we found that the numbers of *Isl1*^+^ and *Ebf1*^+^ cells were reduced in the LGE SVZ of *Six3-*cKO mice at E16.5 but that there was no significant difference at P0. The accumulation of progenitor cells such as Ascl1^+^ cells in the LGE SVZ of *Six3-*cKO mice may retard differentiation by enhancing the Notching signal. We propose that *Six3* might cell-non-autonomously promote the differentiation of a subpopulation of D1 MSNs that experience delayed differentiation to a certain degree when *Six3* is knocked out in the LGE SVZ.

*Six3* was mainly expressed in precursor cells and newborn immature D2 MSNs in the LGE SVZ. Loss of *Six3* function in progenitor cells resulted in a significant reduction in the number of mature D2 MSNs, whereas *Six3*-knockout in differentiated D2 MSNs (*Drd2*^+^) had little effect on striatal MSN development. This indicates that the functions of *Six3* decrease as D2 MSNs mature. The increased apoptosis in the absence of *Six3* may be because *Six3* is required for the survival of immature D2 MSNs, since SIX3 is expressed in immature D2 MSNs. However, in *Drd2-cre*, *Six3*^F/F^ mice, in which *Six*3 was deleted in immature D2 MSNs, the number of D2 MSNs was comparable to that of control mice. This demonstrates that *Six3* plays a minor role in immature D2 MSN survival. Apart from that, large numbers of SP9^+^, *Six3OS*^+^ putative D2 MSNs were found in the LGE SVZ and MZ of *Six3-*cKO mice. But few *Drd2*^+^, *Adora2a*^+^ immature D2 MSNs were observed. This suggests that the differentiation of precursor cells is blocked in the absence of *Six3*. Thus, the increased apoptosis and reduction in the number of D2 MSNs in the absence of *Six3* occurs mainly in response to the abnormal differentiation of D2 MSNs.

Here, we found that *Six3OS* was also co-expressed with *Six3* in the LGE SVZ but down-regulated in the LGE MZ. *Six3* was strongly expressed in the LGE SVZ and its expression was scattered in the LGE MZ. The difference in expression patterns between *Six3* and *Six3OS* indicates that they have different functions in LGE development. The *Six3OS*/*Six3* co-expression pattern in the LGE SVZ indicates that *Six3OS*^+^/*Six3*^+^ cells are precursor cells and that *Six3OS*^*−*^/*Six3*^+^ cells are differentiated D2 MSNs. Thus, the accumulation of many *Six3OS*^+^ progenitor cells in the LGE SVZ of *Six3-*cKO mice suggests that D2 MSN differentiation was blocked due to the loss of *Six3*; however, the migration of D2 MSNs was less affected in *Six3-*cKO mice, as we found that many *Six3OS*^+^ and BCL11B^+^/EBF^*−*^ cells were distributed in the LGE MZ.

It is noteworthy that the numbers of *Drd2*^+^ and *Adora2a*^+^ cells in the LGE MZ at E16.5 and P0 were significantly fewer than those in the *Six3-*cKO striatum at P11, indicating that a small population of precursor cells differentiates into mature D2 MSNs postnatally. We hypothesize that these mature D2 MSNs are largely generated in the pLGE4, as *Six3* is most prominently expressed in the pLGE3. Thus, we propose that most D2 MSNs are generated in the pLGE3 and that a small number of D2 MSNs are derived from the pLGE4. Whether D2 MSNs with different origins exhibit different axonal guidance and functions requires further study. Our RNA-seq data showed that expression of *Six2*, a homologous gene of *Six3*, in the LGE was significantly up-regulated in *Six3-*cKO mice compared to control mice (data not shown). This suggests that *Six2* may have functional redundancy with *Six3* and in turn partly promotes the generation of a subset of D2 MSNs.

In summary, in this study, we provide evidence that *Six3* is an important regulatory element in the LGE SVZ, where it specifically promotes the differentiation of D2 MSN precursor cells. Ongoing studies are aimed at elucidating the molecular mechanisms underlying the distinct functions of *Six3*. These findings broaden our comprehension of the transcriptional mechanisms underlying the development of striatal projection neurons.
